# 

**Published:** 1896-06

**Authors:** 


### CONTENTS.

### COMMUNICATIONS.

The Electrical Aspect of Cataphoresis.................................... 261
Dental Vulcanite.................................... 268
The X-Rays in Dentistry—A Whimsey.................................... 272
A Porcelain Inlay Ground to Fit a Cavity in a Tooth.................................... 277
Dental Caries during Pregnancy.................................... 280
Michigan State Board of Examiners in Dentistry.................................... 282

### SELECTIONS.

Population of Greater New York.................................... 276
How Carborundum is Made.................................... 285
Role of the Nerves in the Mind Cure.................................... 288
Relief for Bee-Stings.................................... 289
A Blow at the Germ Theory.................................... 290
Baby's Rights.................................... 290
What is the Possible Minimum Death Rate?.................................... 291
The Hygienic Importance of Amusements.................................... 291
The Offiicial Languages at the International Medical Congress at Moscow in 1897.................................... 292
Second Pan-American Medical Congress.................................... 292
Medical College Association.................................... 292
Ohio's Tobacco Law.................................... 292
Heroic Treatment for Drunkards.................................... 293
The Retro-Buccal Method of Exposing the Third Branch of the Trigeminus.................................... 293
Higher Medical Education.................................... 294
A Case of Unusual Speech Defect.................................... 294
How to Sterilize Cotton.................................... 295
Treatment of Alcoholism by Strychnine Nitrate.................................... 295
Brandy Drops.................................... 295
Insomnia.................................... 296
Aluminum in the United States.................................... 296
A New Dressing in Surgery.................................... 296
How Austria Deals with Drunkards.................................... 297
Care of the Hair.................................... 297
Composition of Physiology.................................... 298
Slobbering.................................... 298
Good Breeding.................................... 299
Chloroform Anæsthesia Dangerous to Meat-Eaters.................................... 299
Onions.................................... 299
Nutritive Material.................................... 300

### COMMENCEMENTS.

Missouri Dental College.................................... 300
University of Maryland—Department of Dental Surgery.................................... 301
Chicago Northwestern University—Dental Department.................................... 301
University of Buffalo—Dental Department.................................... 302
Cincinnati Academy of Dentistry.................................... 302
Indiana Dental College.................................... 303
Baltimore College of Dental Surgery.................................... 303
Cleveland University of Dentistry and Surgery—Dental Department. 304
Philadelphia Dental College.................................... 304
Vanderbilt University—Dental Department.................................... 306
Birmingham Dental College.................................... 306
Pennsylvania College of Dental Surgery.................................... 307
University of Iowa—Dental Department.................................... 308
Meharry Medical College—Dental Department.................................... 308
Kansas City Dental College.................................... 309

### EDITORIAL.

Change of Place.................................... 309
Inter-State Dental Meeting.................................... 310
The Odontological Society.................................... 311
The American Academy of Dental Science.................................... 311
In Memory of the Late Dr. W. H. Dwinelle.................................... 312
Notice.................................... 312

